# The reliability, validity and screening effect of the happiness index scale among inpatients in a general hospital

**DOI:** 10.1186/s12888-022-04219-0

**Published:** 2022-09-09

**Authors:** Yizhong Shen, Shuai Yuan, Jingwen Liu, Bin Sun, Zilin Chen, Lijiao Zheng, Lihao Chen, Hanwei Chen, Huiqiang Feng, Hongbo He

**Affiliations:** 1 The Affiliated Brain Hospital of Guangzhou Medical University, Guangzhou, China; 2 The Third People’s Hospital of Zhuhai, Zhuhai, China; 3 Guangzhou Panyu Central Hospital, Guangzhou, China

**Keywords:** Happiness Index Scale, Reliability, Validity, Psychological Screening, General Hospital

## Abstract

**Background:**

The Happiness Index Scale (HIS) is a newly developed scale by our group to screen for common psychological illnesses among general hospital inpatients. This study aimed to analyze the reliability, validity and screening effect of the HIS and to explore its clinical application.

**Methods:**

From April 1, 2021, to December 31, 2021, a total of 8405 continuous inpatients were enrolled from different departments of a large tertiary general hospital with 1385 inpatient beds in Guangzhou, Guangdong Province, China. Using a cross-sectional survey design, each participant was assessed with the Patient Health Questionnaire 9(PHQ-9), Generalized Anxiety Disorder 7 items(GAD-7), Athens Insomnia Scale (AIS), Columbia Suicide Severity Rating Scale (C-SSRS) and HIS within 24 h of admission. McDonald's ω coefficient, the Guttman split-half coefficient and the test–retest reliability coefficient were used to evaluate the reliability of the HIS and the construct validity and criterion validity of the validity tests. Scores on the PHQ-9, GAD-7, AIS, and C-SSRS were used as the gold standard tools to analyze the screening effect of the HIS.

**Results:**

The HIS exhibited very good reliability, with a McDonald's ω coefficient of 0.825, a Guttman split-half coefficient of 0.920 and a test–retest reliability coefficient of 0.745 (*P* < 0.05). Confirmatory factor analysis showed a satisfactory model fitting index with a χ^2^/df = 2.602, a root mean squared error of approximation (RMSEA) of 0.014, a standardized root mean square residual (SRMR) of 0.010, a comparative fit index (CFI) of 0.992, and a Tucker–Lewis index (TLI) of 0.983. The correlation coefficient between the total score of each dimension of the scale and the corresponding criterion was 0.854 ~ 0.949 (*P* < 0.001). The HIS showed a very good distinguishing effect. The average HIS score of inpatients who screened positive for psychological problems was significantly higher than that of inpatients who screened negative for psychological problems (*t* = 3790.619, *P* < 0.001). The effect size was very large (Cohens *d* = 2.695, 95% *CI* = 2.630 ~ 2.761). Approximately 90.2% of the positive and negative screening results of the HIS were matched with the gold standard tools, with a kappa value of 0.747 (*P* < 0.001). The screening effect test showed a sensitivity (true positive rate) of 92.9% and a specificity (true negative rate) of 89.5%.

**Conclusion:**

The HIS exhibited satisfactory reliability and validity and a clinically meaningful screening effect with a much shorter version compared to the commonly used screening scales. Thus, it could potentially be useful as the first screening step to rule out psychological conditions for inpatients in general hospitals or to remind medical teams of further psychological concerns.

**Supplementary Information:**

The online version contains supplementary material available at 10.1186/s12888-022-04219-0.

## Background

The deterioration of physical health often causes great psychological pressure, and mental health problems are common in individuals with physical illness in the general population [1–4]. Studies have shown that the prevalence rate of mental disorders is significantly higher in general hospital inpatients with relatively critical and complex physical illnesses (43.7%) than in physically healthy subjects (25.0%) [5]. In China, at least 17.3% of medical and surgical outpatients are reported to have at least one psychiatric disorder [6]. The results of Kroenke's study found that depression and anxiety disorders were the most prevalent psychological disorders, both in general hospital and specific patient groups, with a prevalence of at least 5 to 10% and frequent comorbidities [7]. And depression and anxiety disorders are highly correlated with insomnia [8]. Meanwhile, mental illness can exacerbate or worsen an existing physical illness, lead to slow recovery [9, 10], and increase the risk of social dysfunction and suicide [11–13]. According to some statistics, 43% of the individuals who commit suicide in China suffered from serious physical diseases [14]. We screened 916 inpatients from a large tertiary general hospital for 2 days and found that 37.0% of the inpatients had at least one psychological problems, including 23.8% with depression, 15.4% with anxiety, 28.1% with insomnia and 5.4% with suicide risk [15]. As mentioned above, psychological problems such as depression, anxiety, insomnia and suicide risk are prevalent among inpatients in general hospitals.

Although mental illness is prevalent among general hospital inpatients, most general hospitals in China do not yet have psychiatric departments [16]. It has been reported that nonpsychiatrists in general hospitals usually pay more attention to physical diseases when diagnosing and treating patients, and they often ignore mental health problems, resulting in a generally low recognition rate of psychological disorders among general hospital inpatients [17]. A screening of depression and anxiety disorders in 15 general hospitals in China showed that about 16.5% of inpatients were screened for depression or anxiety disorders, of which only 8.5% were recommended for psychiatric consultation, only 6.4% received psychiatric pharmacological interventions, and most patients (80.8%) received only routine management of their own somatic diseases [18]. Screening of depressive disorders in outpatient internal medicine departments of 23 general hospitals in Shenyang showed that approximately 11.0% of patients were screened for co-morbid depressive disorders, of which only 4.0% were diagnosed by clinicians and only 3.0% were treated with antidepressant medication [19]. These surveys showed that non-psychiatrists in Chinese general hospitals had significantly lower recognition rates of mental disorders than in Western countries (32.5–64.3%) [20–22].

Meanwhile, the somatic symptoms of mental disorders may mask the subjective manifestations of the original mental disorder [23]. It has been reported that more than two-thirds of patients with depression and/or anxiety disorders initially visit general hospitals only for somatic symptoms such as dizziness, headache, palpitations, chest pain, fatigue, insomnia and abdominal pain [24], resulting in a high rate of misdiagnosis, as patients with mental disorders are easily misdiagnosed as having other organic diseases. A study on the misdiagnosis of psychiatric disorders in general hospitals investigated 1062 patients with psychiatric disorders who had been seen in general hospitals. The results showed that 45% of the study subjects were initially seen in other nonpsychiatric outpatient clinics, with a misdiagnosis rate of 42% in the full sample; 6.3% of the patients had been hospitalized due to misdiagnosis, with 40% of them being hospitalized more than twice [25]. Therefore, most nonpsychiatric department inpatients have mental illnesses that are left untreated or misdiagnosed, which not only wastes medical resources but also adds to the financial and mental burden of patients while causing tension between doctors and patients [26].

On the other hand, a shortage of psychiatric professionals in general hospitals in China is common, with only 43.19% of secondary and tertiary general hospitals having a psychiatric department [16]. There is a relative lack of psychiatric consultations in general hospitals, and one study reported a psychiatric consultation rate of 0.6 ~ 1.26% in general hospitals in China [27–29], which is lower than that reported to be approximately 2.6 ~ 3.3% in western countries [30, 31]. For general hospital patients with comorbid psychiatric disorders, general hospital nonpsychiatrists are often their primary care physicians. However, nonpsychiatrists in general hospitals lack practical experience in the diagnosis and treatment of psychiatric disorders and are particularly unskilled at identifying patients with psychiatric disorders with significant somatic symptoms [32]. As a result, such patients are often underdiagnosed or misdiagnosed, leading to delays in treatment. Such delays can lead to a number of problems, the most serious of which is an increased risk of suicide.

The identification and treatment of mental disorders are receiving increasing attention in China. The "Health China 2030 Planning Outline" document proposes that establishing an effective screening and intervention system for known high-risk groups and improving the capacity of mental health services in medical institutions are the pressing key tasks [33]. In 2021, Guangdong Province officially piloted the "Guangdong Happy Hospital (GHH)" project, which aims to improve the early recognition rate of mental disorders among inpatients, and to improve the mental health service capacity of comprehensive medical institutions [34]. However, psychiatrists in general hospitals have limited resources for conducting a psychological assessment interview for each inpatient [16]; therefore, the initial intention of our project was to develop the HIS as an initial screening tool and to complete an initial screening of each patient prior to hospitalization by the HIS within 1 min. After the patients who screened positive entered the ward, the technically trained "Happiness nurse" conducted a detailed interview and psychological assessment and ultimately performed graded and classified specialty interventions based on the assessment results.

The psychological assessment scales are important tool for initial screening to identify a patient’s mental health status, it has the advantages of objective results, quantitative description, clear rating, and economic convenience [35]. Patients with psychological distress may perceive less evident stigma when reporting their problem to the physicians. It also could help the non-psychiatric physicians pay more attention to the psychological distress in limited time and manage it in suggested procedure according to the results [36]. However, the current problem is that most psychiatric self-assessment scales only cover one assessment dimension; to assess a patient’s mental health status in multiple dimensions, multiple scales need to be used together, which can lead to a large number of items measured by patients and take a long time, which is inappropriate for busy clinical staff and anxious patients [36, 37]. There are relatively few reports on whether the multi-dimensional scales in foreign countries are applicable to general hospitals in China, and it may be influenced by cultural differences in the interpretation and expression of scale content that are difficult for Chinese patients to understand [38]. Based on these issues and combined with the prevalence of depression and anxiety, insomnia and suicide risk in general hospital patients, we extracted items from the different dimensional scales available that are internationally recognized and applicable to clinical screening in China. Namely, the PHQ-9, GAD-7, AIS and C-SSRS [39–42], and developed a concise primary screening scale, the HIS, which has multiple dimensions, fewer entries, and fewer time-consuming features [34]. The scale includes 8 items covering 4 factors: depression, anxiety, insomnia and suicidality. The development of the HIS allows for rapid initial screening to identify general hospital inpatients with possible psychological problems to provide a basis for subsequent comprehensive psycho-physiological assessment and intervention by licensed psychiatrists for inpatients who screen positive for psychological problems and improving the scarcity of psychiatric resources.

In a previous study, we validated the HIS with good reliability and validity by analyzing psychometric data from 458 nonpsychiatric inpatients in Guangzhou, China [34]. For subsequent application at a larger scale, in this study, we expanded the sample size to include all inpatient units in a large tertiary general hospital in Guangzhou, China, to assess the reliability and validity of the HIS using psychometric data from 8405 nonpsychiatric inpatients to provide a more robust basis for the use of the HIS for the clinical screening of inpatients in general hospitals and to verify its feasibility in different clinical departments.

## Materials and methods

### Study sites and participants

The study was conducted in Guangzhou Panyu Central Hospital, a 1385-bed tertiary general hospital in Guangzhou, Guangdong Province, which is the 3^rd^ largest city in China, with a population of approximately 127 million. The inclusion criteria were: patients with a clear consciousness and understanding of the contents of the questionnaire; patients who provided informed consent for voluntary participation in this survey. The exclusion criteria were: patients who suffered from serious physical diseases and were unable to complete the questionnaire; patients who were undergoing surgery. A total of 9038 inpatients were enrolled, including 8405 valid questionnaires. This study was approved by the Ethics Committee of the Affiliated Brain Hospital of Guangzhou Medical University and Guangzhou Panyu Central Hospital. Each patient was provided with an electronic informed consent after the researcher was informed in person of the content and purpose of the study.

### Data collection

Twenty-eight nurses from 28 inpatient wards participated in this study, and each of the nurses was responsible for data collection from their ward in Guangzhou Panyu Central Hospital. All nurses received 12 h of face-to-face course training, including training on project introductions, ethical issues of the survey, psychological assessments, common psychological symptoms and common psychiatric diseases, common psychological intervention methods, etc. After patient admission, the participating nurse informed the patient of the content and purpose of the research project. Under instruction, the patient scanned the project bar code with their own cell phone to enter the “questionnaire star” application. If the patient agreed to participate in the survey, they clicked the informed consent option and proceeded to the formal answer interface; if the patient did not agree to participate, they clicked the reject answer option and exited the application. The survey was conducted in a relatively quiet independent ward. All patients answered the questionnaires within 24 h of admission, and the evaluation took 10 ~ 15 min. In addition, 87 randomly selected participants were evaluated with the same scale again after a 2–3 week inpatient stay for the test–retest reliability test.

### Assessment tools

The sociodemographic data including gender, age, marital status, education level, monthly income and inpatient department of all the participants were collected.

The HIS scale [34]: The HIS is obtained by extracting 8 items from 31 items in PHQ-9, GAD-7, AIS and C-SSRS by GHH project team through exploratory factor analysis [34]. The psychometric properties of the scale have been initially validated in a sample of 458 cases [34]. The scale covers 4 factors, each containing 2 entries. The eigenvalues of the four factors ranged from 0.79 to 3.30 and the four factors explained 84.2% of the total variance in the results. The factor loading of each entry is greater than 0.8. Factor 1 includes 2 items from the PHQ-9 related to the core items of depression(HIS-1: Little interest or pleasure in doing things; HIS-2: Feels down, depressed, or hopeless). Factor 2 includes 2 items from the GAD-7 related to anxiety(HIS-3: Worries too much about different things; HIS-4: Becomes easily annoyed or irritable). Factor 3 includes 2 items from the AIS related to sleep quality(HIS-5: Total sleep time; HIS-6: Total sleep quality (no matter how long you sleep). Factor 4 includes 2 items from the C-SSRS related to suicidal thoughts(HIS-7: Do you actually have some thoughts about suicide?; HIS-8: Have you been thinking about how to kill yourself?). Among the 8 items of the scale, Items 1 ~ 6 are scored with 0 ~ 3 points (0 = "never", 1 = "a few days", 2 = "more than half the days", 3 = "almost every day"), and Items 7 ~ 8 are answered with "yes" or "no". Each score is multiplied by the corresponding weight and summed to obtain the actual score of the scale. See additional file 1 for details on how weights are calculated. Plotting ROC curves to determine cutoff values of scale scores [34]. The total score of the final scale ranges from 0 ~ 6.247. A total score of 0 ~ 0.364 indicates no psychological problems, 0.365 ~ 0.954 indicates mild psychological problems, 0.955 ~ 1.469 indicates moderate psychological problems, and 1.470 ~ 6.247 indicates severe psychological problems. See Table 1 for details.

**Table 1:** Happiness index scale (HIS)

**Items**	PCA	**Score**			
	Loading	never	a few days	more than half the days	almost every day
**Factor 1: Depression trend**					
HIS-1 (PHQ-1): Little interest or pleasure in doing things	0.879	0	1	2	3
HIS-2 (PHQ-2): Feels down, depressed, or hopeless	0.811	0	1	2	3
**Factor 2: Degree of anxiety**					
HIS-3 (GAD-3): Worries too much about different things	0.822	0	1	2	3
HIS-4 (GAD-6): Becomes easily annoyed or irritable	0.854	0	1	2	3
**Factor 3: Sleep quality**					
HIS-5 (AIS-4): Total sleep time	0.913	0	1	2	3
HIS-6 (AIS-5): Total sleep quality (no matter how long you sleep	0.902	0	1	2	3
		no		yes	
**Factor 4: Suicidal tendency**					
HIS-7 (C-SSRS-2): Do you actually have some thoughts about suicide?	0.921	0		1	
HIS-8 (C-SSRS-3): Have you been thinking about how to kill yourself?	0.930	0		3	

HIS actual score = 0.203 × HIS1 + 0.169 × HIS2 + 0.143 × HIS3 + 0.177 × HIS4 + 0.297 × HIS5 + 0.326 × HIS6 + 1.021 × HIS7 + 0.427 × HIS8

The entries of the HIS were extracted from a total of 31 entries of the PHQ-9, GAD-7, AIS, and C-SSRS. The PHQ-9, GAD-7, AIS and C-SSRS were used as the criteria to analyze the criterion validity of the HIS. The specificity and sensitivity of the HIS were calculated using the above four classical self-assessment scales as the "gold standard".

The evaluation result of the "gold standard" was defined as follows: If at least one of the abovementioned four classical self-assessment scales had mild results or higher, it represented positive results for psychological problems in the "gold standard" evaluation. The HIS rated psychological problems as mild or higher as indicative of positive results for psychological problems.

### Statistical analysis

Based on the factor structure of the exploratory factor analysis in the previous development of the HIS, this study used a larger sample size to test the structural validity of the scale through confirmatory factor analysis. Pearson correlation analysis was used to test the criterion validity of the scale. The intergroup difference in the HIS average score of the inpatients who screened positive or negative for psychological problems by the "gold standard" was tested by the Welch's robust T-Test. Descriptive parameters using Cohen’s *d* for indicating effect size were used. McDonald's Omega and the Guttman split-half coefficient were used to test the internal consistency reliability of the scale, and Pearson correlation analysis was used to test the test–retest reliability of the scale. The sensitivity and specificity of the HIS were calculated by screening effect analysis. *P* < 0.05 indicated that the difference was statistically significant. Descriptive statistics were computed utilizing the statistical software IBM SPSS Statistics 22.0 while confirmatory factor analysis was conducted with the R package Lavaan.

## Results

### General demographic data

The age of inpatients was mostly concentrated 18–59 years old (5348 patients; 63.6%), and there were more female inpatients than male inpatients (4420 patients; 52.6%). The education level of the inpatients was low, and 61.6% of the subjects had been educated for 9 years or less. Married inpatients significantly outnumbered unmarried inpatients. Slightly more inpatients earn less than 5,000 RMB per month. All subjects were from nonpsychiatric departments, of which there were more inpatients in the Surgery department (42.5%; see Table 2 at the end of the article for details).

**Table 2:** General demographic data of the inpatients (*n* = 8405)

Project	Total sample (*n* = 8405)	Mean	SD	E	*95%CI*		Min	Max	*Welch*	*P*
					Min.limit	Max.limit				
Gender									67.722	< 0.001
Male	3985 (47.4)	0.290	0.564	0.010	0.273	0.308	0.000	5.310		
Female	4420 (52.6)	0.400	0.662	0.010	0.381	0.420	0.000	5.481		
Age									8.011	< 0.001
≤ 17 years old	149 (1.8)	0.305	0.684	0.056	0.194	0.415	0.000	5.481		
18–59 years old	5348 (63.6)	0.367	0.626	0.008	0.350	0.383	0.000	5.481		
≥ 60 years old	2908 (34.6)	0.313	0.606	0.011	0.291	0.335	0.000	5.310		
Education (years)									29.991	< 0.001
≤ 9	5175 (61.6)	0.319	0.611	0.009	0.302	0.335	0.000	5.481		
> 9	3230 (38.4)	0.395	0.631	0.011	0.374	0.417	0.000	5.310		
Marital status									30.388	< 0.001
Married	7265 (86.4)	0.331	0.602	0.007	0.318	0.345	0.000	5.310		
Unmarried	1140 (13.6)	0.455	0.717	0.021	0.413	0.497	0.000	5.481		
Monthly income									0.303	0.582
Less than 5,000 RMB	4667 (55.5)	0.352	0.644	0.009	0.333	0.370	0.000	5.481		
More than 5,000 RMB	3738 (44.5)	0.344	0.589	0.010	0.325	0.363	0.000	5.318		
Inpatient department									28.203	< 0.001
Internal medicine	3068 (36.5)	0.387	0.660	0.012	0.364	0.410	0.000	5.481		
Surgery	3575 (42.5)	0.283	0.579	0.010	0.264	0.302	0.000	4.932		
Obstetric/Gynecology	1356 (16.1)	0.438	0.613	0.017	0.406	0.471	0.000	4.966		
Ophthalmology/Otolaryngology	406 (4.9)	0.328	0.625	0.031	0.267	0.389	0.000	5.318		

Welch's T-test showed that inpatients with female (0.400 ± 0.662), more than 9 years of education (0.395 ± 0.631) and unmarried (0.455 ± 0.717) had relatively higher HIS scores. Multiple comparisons showed that the HIS scores of inpatients in internal medicine (0.387 ± 0.660) and obstetrics/gynecology (0.438 ± 0.613) were higher than those in surgery (0.283 ± 0.579), and those in obstetrics/gynecology were higher than those in ophthalmology/otolaryngology (0.328 ± 0.625). HIS scores of inpatients aged 18–59 years (0.367 ± 0.626) were higher than those aged 60 years or older (0.313 ± 0.606).

### Validity evaluation results of the HIS

#### Construct validity

**Fig. 1:**
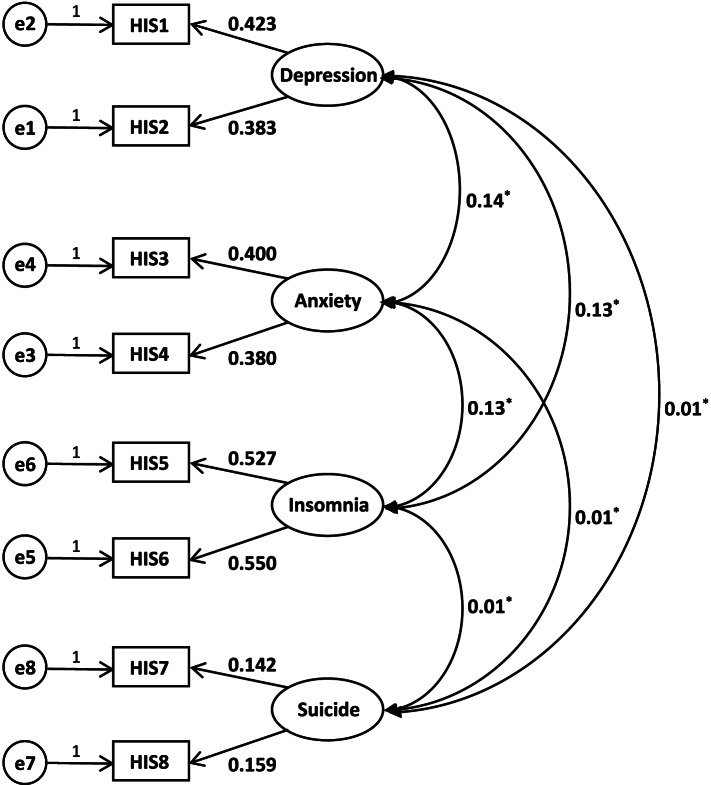
4-factor structural equation model. Note. Parameter estimation methods for confirmatory factor analysis using weighted least squares means and variances (WLSMV). The loadings in the figure are normalized. All loadings in figure are significative. *p < .001

Factor structure based on exploratory factor analysis from the results of the development of the HIS [34]. This study conducted confirmatory factor analysis to evaluate the structural validity based on 8405 study participants. With eight entries as observed variables and four factors as latent variables, the loadings for each entry and the correlations between the factors are shown in Fig. 1, with the factors significantly uncorrelated (*r* = 0.01–0.14, *P* < 0.001). The fitting index results of the various models showed a *χ*^*2*^/df of 2.602, an RMSEA of 0.014, a CFI of 0.992, a TLI of 0.983, and a SRMR of 0.010. The results showed that the fit index of the model was satisfactory. It can be concluded that the scale has high structural validity (see Fig. 1 for the structural equation model).

#### Criterion validity

As described in the Methods section, the HIS included 4 factors (depression, anxiety, insomnia and suicide), with 2 items for each factor. The PHQ-9, GAD-7, AIS and C-SSRS were used as the criteria to predict the criterion validity of the HIS. Pearson correlation analysis showed that the total score of each factor of the HIS was highly positively correlated with the total scores of the corresponding classical scales. The correlation coefficients were as follows: depression: *r* = 0.854; anxiety: *r* = 0.935; insomnia: *r* = 0.921; and suicide: *r* = 0.949 (*P* < 0.001). The details are shown in Table 3.

**Table 3:** Correlation between HIS factors scores and classical scale scores

Factor	HIS factor score (M ± SD)	Classical scale score (M ± SD)	*r*	*p*
Depression	0.41 ± 0.904	1.69 ± 3.227	0.854	< 0.001
Anxiety	0.34 ± 0.864	1.01 ± 2.584	0.935	< 0.001
Insomnia	0.62 ± 1.141	2.27 ± 3.679	0.921	< 0.001
Suicide	0.04 ± 0.354	0.12 ± 1.192	0.949	< 0.001

HIS factor score is the pre-weighting score

#### Distinguishing effect

The "gold standard" screened 1896 inpatients with psychological problems and a mean HIS score of ( 1.207 ± 0.776); 6509 inpatients without psychological problems and a mean HIS score of ( 0.098 ± 0.208). The Welch's robust T-Test showed that inpatients with psychological problems had significantly higher HIS scores than those without psychological problems (*t* = 3790.619, *P* < 0.001). The effect size was very large (Cohens *d* = 2.695, 95% *CI* = 2.630 ~ 2.761). The results showed that the HIS could clearly distinguish between inpatients with and without psychological problems, indicating a high distinction effect of the HIS. Descriptive statistics were done for each item, which includes mean, standard deviation, measures of skewness and kurtosis as shown in Table 4.

**Table 4:** Descriptive statistics for items (*n* = 8405)

Item	M	SD	Min	Max	Skewness	Kurtosis
HIS 1	0.047	0.108	0.000	0.609	2.757	8.736
HIS 2	0.030	0.077	0.000	0.507	2.986	10.704
HIS 3	0.026	0.069	0.000	0.429	3.219	11.892
HIS 4	0.029	0.081	0.000	0.531	3.339	13.119
HIS 5	0.090	0.179	0.000	0.891	2.128	4.474
HIS 6	0.104	0.196	0.000	0.978	1.977	3.843
HIS 7	0.013	0.113	0.000	1.021	8.824	75.876
HIS 8	0.010	0.111	0.000	1.281	11.331	126.412

### Reliability evaluation results of the HIS

The McDonald's omega of the HIS were 0.825, and the McDonald's omega for each factor ranged from 0.798 ~ 0.891 (*P* < 0.05), indicating that the items of the scale had high internal consistency.

The divided half reliability coefficient model was used to analyze the scale, and the items were randomly divided into two groups. One group comprised the HIS-1, HIS-3, HIS-5 and HIS-7. The other group comprised the HIS-2, HIS-4, HIS-6, and HIS-8. The results showed that the Guttman split-half coefficient of the HIS was 0.920, and that of each factor ranged from 0.720 ~ 0.891 (*P* < 0.05), indicating that the Guttman split-half coefficient of the scale was high.

Eighty-seven randomly chosen patients were evaluated with the HIS twice (with an interval of 2–3 week) to evaluate the test–retest reliability. The results showed that the correlation coefficient of the total scores of the two groups was 0.745, and the correlation coefficient of the scores of the two groups for each factor ranged from 0.640 ~ 0.863 (*P* < 0.05). The test–retest correlation coefficient of each test score of the HIS showed that the consistency between the retest score and the initial test score was high, and the stability of the HIS was high (see Table 5 for details).

**Table 5:** Reliability test of the HIS and each dimension

Reliability statistic				
Dimension	Number of items	McDonald's ω(*n* = 8405)	Guttman Split-Half (*n* = 8405)	Test–retest reliability (*n* = 87)
Depression	2	0.798	0.792	0.809
Anxiety	2	0.815	0.814	0.863
Insomnia	2	0.891	0.891	0.800
Suicide	2	0.878	0.720	0.640
Total	8	0.825	0.920	0.745

One Correlation Power Analysis: A sample size of 86 achieves 90% power to detect a difference of 0.20000 between the null hypothesis correlation of 0.75000 and the alternative hypothesis correlation of 0.55000 using a two-sided hypothesis test with a significance level of 0.05000

The demographic distribution of the 87 retest samples was not significantly different from the total sample. See additional file 1 for details

### Analysis of the HIS screening effect

Screening with the HIS detected 5959 (70.9%) patients without psychological problems and 2446 (29.1%) patients with psychological problems. Compared with "gold standard" screening, "gold standard" detected 6509 (77.4%) patients without psychological problems and 1896 (22.6%) patients with psychological problems. A screening effect analysis was conducted, and indicators of the accuracy of screening with the HIS were compiled. The screening consistency rate between the HIS and "gold standard" was 90.2% [(5824 + 1761)/8405], and the kappa value was 0.747 (*P* < 0.01). The HIS assessment was in high agreement with the "gold standard" (see Table 6). The sensitivity (true positive rate) of the HIS was 92.9%, and the specificity (true negative rate) was 89.5%. The missed diagnosis rate (false negative rate) was 7.1%, and the misdiagnosis rate (false-positive rate) was 10.5%. The positive predictive value (PPV) was 72.0%, and the negative predictive value (NPV) was 97.7%.

**Table 6:** Analysis of the HIS screening effect

		Gold standard		Total
		Negative	Positive	
HIS	Negative	5824	135	5959
	Positive	685	1761	2446
	Total	6509	1896	8405

## Discussion

This study used data from 8405 non-psychiatric inpatients in Guangzhou, China to evaluate the reliability, validity and screening effect of HIS. Extensive evaluation showed that this scale has satisfactory reliability and validity as well as screening effects. Meanwhile, depression and anxiety disorders are prevalent and highly comorbid in general hospitals, with a greater chance of insomnia and suicide risk in severe cases [43, 44]. Therefore, we developed the HIS to obtain a single score about general distress that summarizes the core features of depression and anxiety and includes a high generalization for insomnia (sleep duration and total sleep quality) and two entries regarding suicidal thought. The HIS thus reasonable based on its content. It is also an improvement on all of the above issues, as reflected by the inclusion of multiple dimensions, a smaller number of entries, and simplicity of use.

Our group conducted an exploratory factor analysis on data from 458 previous patients [34], and considering that the scale needs to contain 4 dimensions common in general hospitals (depression, anxiety, insomnia and suicide risk). Therefore, the number of factors was set to four before exploratory factor analysis, and four factors with eigenvalues in the range of 0.79–3.30 were obtained. Usually the factor eigenvalues need to be greater than 1, but the cumulative variance contribution of the four factors was 84.2%, and the factor loadings of all eight entries were above 0.8, which generally met the statistical criteria. In this study, based on the structure of the four factors obtained from the exploratory factor analysis in the above development results [34], a validation factor analysis was conducted, and the structural equation model showed that the four factors were independent of each other (*r* = 0.01–0.14, *P* < 0.001) and the structural model equation fitted well, indicating satisfactory structural validity of the scale. Figure 1 shows that the depression factor was not significantly correlated with the suicide factor, which may be due to the fact that general hospital inpatients are non-psychiatric inpatients and the risk of suicide may be related to a variety of factors such as their own serious physical illness, the social environment in which they live, or other psychiatric symptoms [45–52]. However, the original purpose of the HIS was rapid primary screening, and it was not possible to include various risk factors in the scale, so this scale includes suicide as a separate factor to screen patients for psychological problems in order to provide a basis for a comprehensive assessment by subsequent professionals. The correlation between HIS and PHQ-9, GAD-7, AIS and C-SSRS further demonstrated the satisfactory criterion validity of the HIS (*r* = 0.854–0.949, *P* < 0.001). The average HIS score of inpatients with psychological problems was significantly higher than that of inpatients without psychological problems (*t* = 3790.619, *P* < 0.001), and the effect size was very large (Cohens *d* = 2.695, 95% *CI* = 2.630 ~ 2.761); that is, the distinction effect of the HIS was very good, and it can effectively distinguish inpatients with psychological problems from inpatients without psychological problems. In summary, inpatients with psychological problems can be more clearly and accurately identified by psychiatrists through HIS, which is convenient for subsequent timely psychological evaluations and graded interventions and avoids the waste of medical resources.

To support construct validity, an analysis of variance was applied to test the interrelations among the psychological health level and socio-demographic variables. The results showed significant differences in mean HIS scores between the gender, age, years of education, marital status, and disease type (inpatient department) groups. One possible explanation for this difference is that psychological health baseline and psychological resilience levels differ across demographic characteristics. Several surveys in China have found that women, unmarried and middle-aged people have relatively low levels of mental health [53–56]. Meanwhile, the proportion of adult patients in this study sample was large, and there were only 149 (1.8%) patients under 18 years old, so whether HIS is applicable to children and adolescents needs further validation in the future.

In this study, the internal consistency of all 8 items was 0.825, and the internal consistency of the factors was generally higher than the accepted value of 0.70. The Guttman split-half coefficient was 0.920, which also proved that the scale has high reliability. There were 87 participants who were administered the HIS twice (2–3 weeks apart) to evaluate the test–retest reliability. The correlation coefficient of the two scores was 0.745 (*P* < 0.001), the consistency and stability of the scale before and after measurement are high. The retest reliability of the suicide dimension was slightly lower, but still acceptable, and the reason considered may be that the risk of suicide in general hospital inpatients changes as their somatic illness improves or worsens [12, 13, 57].

This study analyzed the screening effect of the HIS on inpatients' psychological problems by comparing the evaluation results to the "gold standard". The overall consistency rate between the calculated HIS score and the "gold standard" was 90.2% (Kappa = 0.747, *P* < 0.01)). This shows that the evaluation effect of the HIS is highly consistent with the "gold standard", indicating that the HIS can effectively predict the psychological problems of nonpsychiatric inpatients and that the feasibility of its clinical operation is high. The high true-positive rate and low false-negative rate indicate that the HIS has a high accuracy in screening patients with positive results, which can reduce missed diagnoses in initial screenings and provide timely and effective psychological interventions to high-risk patients accordingly. A higher true-negative rate and a lower false-positive rate reflect the high accuracy of the HIS in narrowing the range of patients with positive results. If the false-positive rate is high, more medical costs and resources will be consumed, and an assessment tool with a high specificity and low misdiagnosis rate is more desirable in current society where mental disorders are highly stigmatized. In conclusion, the evaluation standard of the HIS has high sensitivity and specificity, the corresponding missed diagnosis rate and misdiagnosis rate are low, the accuracy, positive predictive value and negative predictive value results are ideal, and the scale has high diagnostic efficiency.

It is also important to note that because the psychiatric self-assessment scale is hardly the "gold standard" for diagnosing mental disorders, most clinical practices require psychiatrists to conduct the MINI interview with patients as the "gold standard" for diagnosis. However, the limited resources of psychiatrists in general hospitals do not allow them to conduct psychiatric interviews with all inpatients one by one [16], so the HIS scale, as a preliminary screening tool, can easily and efficiently initially screen out inpatients who are more likely to have psychological problems, thus providing a convenient way for subsequent psychiatrists to target patients who screen positive for definitive diagnosis and develop corresponding interventions; this would ultimately improve the identification and treatment rates of psychiatric disorders in general hospitals while avoiding the waste of medical resources and improving the current situation of lack of psychiatrists' resources in general hospitals. Our findings suggest that the HIS is a reliable and valid tool for the initial screening of nonpsychiatric inpatients in general hospitals for psychological problems for clinical and research use.

## Limitations and future direction

This study is subject to several limitations. First, in this study, PHQ-9, GAD-7, AIS and C-SSRS were set as the "gold standard" to analyze the reliability, validity and screening effect of HIS; because the four abovementioned classical self-assessment scales are not 100% accurate and are not the ideal "gold standard", they are slightly less accurate as the "gold standard". Considering the large sample size of the study, the use of clinical diagnostic tools for comprehensive diagnostic assessments was inconvenient and sufficient manpower to conduct a diagnostic "gold standard" for each patient, such as the SCID, the MINI, and face-to-face visits, was lacking. At the same time, the scale was developed with the aim of conducting preliminary psychological problem screening for nonpsychiatric inpatients. Patients screened initially with positive results should be comprehensively evaluated by a psychiatrist with clinical diagnostic tools to achieve the purpose of qualitatively understanding the patients' psychological problems and graded psychological or pharmacological interventions. Second, some of the literature (e.g., Kline 2016 [58] ) suggests that each factor of the scale contains at least three entries. However, for the four factors of the HIS, each factor contains two entries, but in the results of this study, each factor (two entries) score was significantly and highly correlated with the total score of the corresponding criteria (*r* = 0.854–0.949), and the exploratory factor analysis showed that the cumulative variance contribution of the four factors (eight entries) was 84.2%, and the factor loadings of the exploratory factor analysis of all eight entries were above 0.8. Additionally, the HIS is designed to provide an easy and rapid initial screening of psychological problems in general hospital inpatients. Therefore, the impact of each factor (2 entries) on potential problems in this study was relatively small. Finally, because all participants were recruited from a general hospital in Guangzhou, China, caution should be exercised in generalizing the study results to other clinical settings or to the country as a whole. In conclusion, the HIS has satisfactory reliability, validity and screening effect when used for nonpsychiatric inpatients in general hospitals and has high feasibility for psychological illness screening of nonpsychiatric inpatients in general hospitals.

## Conclusion

The HIS exhibited satisfactory reliability and validity and a clinically meaningful screening effect with a much shorter version compared to the commonly used screening scales, and can effectively detect patients requiring further intervention. Thus, it could potentially be useful as the first screening step to rule out psychological conditions for inpatients in general hospitals or to remind medical teams of further psychological concerns. HIS is undoubtedly of great help to the detection rate of psychological problems in general hospitals.

## Supplementary Information

**Additional file 1.** Parameter estimation of linear regression model.

**Additional file 2.** Demographic distribution of the retest sample versus the total sample.

## Data Availability

The datasets used and/or analysed during the current study available from the corresponding author on reasonable request.

## Abbreviations

HIS: Happiness Index Scale
PHQ-9: Patient Health Questionnaire 9
GAD-7: Generalized Anxiety Disorder 7 items
AIS: Athens Insomnia Scale
C-SSRS: Columbia Suicide Severity Rating Scale
RMSEA: Root Mean Squared Error of Approximation
SRMR: Standardized Root Mean Square Residual
CFI: Comparative Fit Index
TLI: Tucker-Lewis Index
GHH: Guangdong Happy Hospital
WLSMV: Weighted Least Squares Means and Variances
