# Correction: The reliability, validity and screening effect of the happiness index scale among inpatients in a general hospital

**DOI:** 10.1186/s12888-022-04264-9

**Published:** 2022-09-22

**Authors:** Yizhong Shen, Shuai Yuan, Jingwen Liu, Bin Sun, Zilin Chen, Lijiao Zheng, Lihao Chen, Hanwei Chen, Huiqiang Feng, Hongbo He

**Affiliations:** 1 The Affiliated Brain Hospital of Guangzhou Medical University, Guangzhou, China; 2 The Third People’s Hospital of Zhuhai, Zhuhai, China; 3 Guangzhou Panyu Central Hospital, Guangzhou, China; 4 Health Commission of Guangdong Province, Guangzhou, China

**Correction: BMC Psychiatry 22, 601 (2022)**

**https://doi.org/10.1186/s12888-022-04219-0**

Following publication of the original article [1], the authors identified that an affiliation was missing. The affiliation has been added and assigned to the author.

Health Commission of Guangdong Province, Guangzhou, China.

The original article [1] has been corrected.
