# Supplementary material for: The reliability, validity and screening effect of the happiness index scale among inpatients in a general hospital
Source: BMC Psychiatry. 2022 Sep 9;22:601. doi: 10.1186/s12888-022-04219-0 (PMC9463772; doi:10.1186/s12888-022-04219-0)
Supplement: Supplementary file 1 — Additional file 1. Parameter estimation of linear regression model. [file 12888_2022_4219_MOESM1_ESM.docx]

Supplementary Material

Entry weights for HIS: With the severity of "gold standard" ^[35]^ as the dependent variable and HIS item score as the independent variable, the linear regression model was fitted for the total score of HIS scale and each item through multiple linear regression analysis, and the model fitting statistics (F=13.745, *P*<0.001). See additional table 1 for details. The parameter estimates of each variable in the model were adjusted appropriately to derive the entry weights: HIS actual Score=0.203×HIS1+0.169×HIS2+0.143×HIS3+0.177×HIS4+0.297×HIS5+0.326×HIS6+1.021×HIS7+0.427×HIS8.

Additional table 1 Parameter estimation of linear regression model

| Item | *B* | *E* | *t* | *P* | *Beta* |
| --- | --- | --- | --- | --- | --- |
| HIS1 | 0.203 | 0.016 | 12.848 | <0.001 | 0.151 |
| HIS2 | 0.169 | 0.019 | 8.769 | <0.001 | 0.108 |
| HIS3 | 0.143 | 0.018 | 8.109 | <0.001 | 0.097 |
| HIS4 | 0.177 | 0.019 | 9.240 | <0.001 | 0.113 |
| HIS5 | 0.297 | 0.017 | 18.006 | <0.001 | 0.246 |
| HIS6 | 0.326 | 0.017 | 19.238 | <0.001 | 0.269 |
| HIS7 | 1.021 | 0.082 | 12.478 | <0.001 | 0.152 |
| HIS8 | 0.427 | 0.037 | 11.697 | <0.001 | 0.142 |
