# Supplementary material for: The reliability, validity and screening effect of the happiness index scale among inpatients in a general hospital
Source: BMC Psychiatry. 2022 Sep 9;22:601. doi: 10.1186/s12888-022-04219-0 (PMC9463772; doi:10.1186/s12888-022-04219-0)
Supplement: Supplementary file 2 — Additional file 2. Demographic distribution of the retest sample versus the total sample. [file 12888_2022_4219_MOESM2_ESM.docx]

Additional table 2 Demographic distribution of the retest sample versus the total sample

| Project | Total sample（%）  (*N*=8405) | Retest sample（%）  (*n*=87) | *χ²* | *P* |
| --- | --- | --- | --- | --- |
| Gender |  |  | 0.003 | 0.958 |
| Male | 3985（47.4） | 41（47.1） |  |  |
| Female | 4420（52.6） | 46（52.9） |  |  |
| Age |  |  | 0.846 | 0.569 |
| ≤17 years old | 149（1.8） | 1（1.1） |  |  |
| 18-59 years old | 5348（63.6） | 60（69.0） |  |  |
| ≥60 years old | 2908（34.6） | 26（29.9） |  |  |
| Education (years) |  |  | 1.002 | 0.317 |
| ≤9 | 5175（61.6） | 49（56.3） |  |  |
| >9 | 3230（38.4） | 38（43.7） |  |  |
| Marital status |  |  | 0.062 | 0.803 |
| Married | 7265（86.4） | 76（87.4） |  |  |
| Unmarried | 1140（13.6） | 11（12.6） |  |  |
| Monthly income |  |  | 0.504 | 0.478 |
| Less than 5,000 RMB | 4667（55.5） | 45（51.7） |  |  |
| More than 5,000 RMB | 3738（44.5） | 42（48.3） |  |  |
| Inpatient department |  |  | 1.449 | 0.694 |
| Internal medicine | 3068（36.5） | 28（32.2） |  |  |
| Surgery | 3575（42.5） | 42（48.3） |  |  |
| Obstetric/Gynecology | 1356（16.1） | 14（16.1） |  |  |
| Ophthalmology/Otolaryngology | 406（4.9） | 3（3.4） |  |  |
